# Perceptions of health and healthcare needs in low-resource settings: qualitative insights from Bengaluru's urban slum and rural areas

**DOI:** 10.3389/fpubh.2025.1530256

**Published:** 2025-04-01

**Authors:** Keerthi Dubbala, Wanda Spahl, Carolin Elizabeth George, Luc de Witte

**Affiliations:** ^1^Department of General Health Studies, Division of Biomedical and Public Health Ethics, Karl Landsteiner University of Health Sciences, Krems an der Donau, Austria; ^2^Department of Community Health, Bangalore Baptist Hospital, Bangalore, Karnataka, India; ^3^The Hague University of Applied Sciences, The Hague, Netherlands

**Keywords:** low-resource settings, resource-limited settings, healthcare, universal health coverage, lay perceptions

## Abstract

**Background:**

Despite the multitude of healthcare services available in India, health inequalities persist. People in low-resource settings are both disadvantaged and have the greatest need for healthcare. To address these disparities and achieve universal health coverage, healthcare services need to be tailored to the specific needs of this population.

**Objective:**

This study aimed to understand health and healthcare perceptions of people in slums and villages in and around Bengaluru, a city in the southern part of India. It was conducted in partnership with Bangalore Baptist Hospital, a charity hospital dedicated to supporting underserved populations in this region.

**Methods:**

The study employed qualitative methods. Twenty-eight open-ended interviews and eight focus groups were conducted with residents of selected slums and villages in and around Bengaluru. The interviews were transcribed verbatim, translated to English and analyzed applying thematic analysis.

**Results and conclusion:**

The study finds that participants defined health as the absence of illness, the ability to work, and the result of a good lifestyle. With regards to healthcare expectations, the analysis shows the themes of the “good doctor,” recovering quickly, cost affordability, cleanliness, and emergency services and diagnostic facilities. In addition, stigma related to healthcare, was identified, especially among residents of villages. Participants highlight the importance of good relationships with healthcare providers and accessible healthcare facilities to improve healthcare uptake in Bengaluru's slums and rural areas. This study also shows that achieving universal health coverage requires addressing not only direct costs but also other associated expenses like travel and lost wages, considering healthcare costs as a comprehensive expense tied to patients' living conditions. These results contribute to the growing body of literature on health and healthcare perceptions in low-resource settings, offering insights that may inform future research and context-specific strategies for improving healthcare access and delivery.

## Introduction

The World Health Organization (1) defines universal health coverage (UHC) as “all people having access to the full range of quality health services they need, when and where they need them, without financial hardship.” This definition includes three main aspects: (1) Equitable access to services for everyone in need, not just those who can afford them, (2) good enough quality of services that improve health, and (3) affordable services that do not impose financial burdens on users (2). For achieving UHC and building effective systems, especially in low-resource settings, it is essential to understand and incorporate people's health perceptions. Often called “lay perspectives,” this area has been widely researched for decades (3–8).

Lay perspectives on health often represent a holistic understanding that emphasizes functionality, participation in society, and a healthy lifestyle, in addition to the absence of illness (3, 5, 8). This holistic view can help explain the incongruence often observed between self-rated health and measurable health status (9, 10). Perceptions of health, influenced by individual and contextual factors such as neighborhood environments (11), social and cultural dynamics (6, 12), psychological aspects (13), and health status, significantly shape health behaviors. These behaviors include lifestyle modifications and treatment adherence, which, in turn, impact health outcomes (4). For instance, positive subjective health perceptions are associated with lower mortality rates in later life stages (9) and improved health-related quality of life and disease management in individuals with cardiovascular diseases and diabetes (6, 14, 15). However, overestimating one's health status has been associated with risky behaviors such as insufficient exercise, poor sleep, unhealthy eating, and daily alcohol consumption, as individuals mistakenly believe they are “healthy enough” to make such choices (16, 17). This complex interplay between subjective health perceptions and health behaviors underscores the need for nuanced approaches to health education and promotion.

In addition to health perceptions, perceptions of healthcare significantly influence care-seeking behaviors and outcomes encompassing factors such as availability, and accessibility, adequacy, effectiveness of services, technical competence of healthcare personnel, patient-provider relationships, and affordability (18–21). Affordability is a critical determinant, with high out-of-pocket costs limiting access, especially in low-income populations. These issues are particularly pronounced in low- and middle-income countries (LMICs), where the limited availability and quality of public healthcare facilities (HCFs) often lead people to rely on informal providers. While these providers are more accessible, concerns about their competence remain (22–26). Moreover, low awareness and limited coverage of public insurance, combined with mistrust in health insurance schemes due to perceived inadequacies in coverage, hinder enrolment (22, 27), further exacerbating access disparities. Additionally, overcrowded clinics and long waiting times exacerbate perceptions of inadequate care, discouraging care-seeking behaviors (23). Interactions with healthcare providers also play a key role in this context. Positive interactions with healthcare providers, characterized by empathy and clear communication, foster trust and encourage care-seeking, while dismissive attitudes, rushed consultations, and skepticism about provider competence act as barriers, particularly in underserved settings (6, 28–30).

We adopt a theoretical approach grounded in the Social Determinants of Health framework, which highlights how personal, economic, social, and geographical factors shape health and healthcare perceptions, and the Health Belief Model, which focuses on how these perceptions and beliefs influence individuals' decisions to assess health services and seek care (31–39). Tackling these challenges is key to improving healthcare services and uptake, ultimately advancing UHC in low-resource settings.

### Low resource settings in India

While India as a whole is classified as an LMIC, certain parts of and communities within India are better regarded as low-resource due to significant resource disparities (40–42). The unequal distribution of healthcare services between rural and urban areas, and within urban regions that is prevalent in India, disproportionately affects these groups (43, 44). About half of the 70% of India's population living in rural areas fall below the poverty line and research shows that their health outcomes are poorer than those living urban regions (45, 46). According to SRS 2018 infant mortality rate of rural population is 50% more than that of the urban population (47). Major barriers to accessing necessary healthcare include distance and poor service quality. Private HCFs, which typically offer better care in India, are concentrated in urban areas (48, 49). Additionally, a large proportion of healthcare providers in rural areas lack formal medical training and are unlicensed (44). Within urban areas, residents of slums face poorer health outcomes compared to those in neighboring areas due to high poverty rates, inadequate sanitation, and geographical isolation from formal institutions, including HCFs (43, 50). Research on LMICs shows that health outcomes tend to even be worse in urban slums than in underserved rural areas. Infant mortality rates are higher (11), and issues such as malnutrition, diarrheal diseases, emotional and behavioral problems in children, and trauma-related deaths are more common (51).

### The Indian healthcare system

The Indian healthcare system is pluralistic, consisting of a combination of private healthcare services and a three-tiered public system that includes primary, secondary and tertiary healthcare (52, 53). Despite the multitude of HCFs available, affordability and accessibility challenges and inadequate public health infrastructure hinder achieving equity in healthcare delivery (19, 25, 44, 49, 52). In India, the overall healthcare expenditure amounts to about 3% of the country's gross domestic product, far below the global average of 10%. Nearly half of this expenditure are out-of-pocket payments by individuals (54). The coverage of the public healthcare system is relatively low, with public services delivering only 8–10% of healthcare, despite being cheaper than private healthcare (55). In contrast, private healthcare services are more widely used, with 70% of urban households and 63% of rural households relying on them (56).

The private healthcare system in India includes both medically qualified healthcare practitioners and indigenous healers using traditional methods such as Ayurveda, Unani, and Homeopathy (57). The infrastructure ranges from small, minimally equipped private practices to multi-specialty hospitals with advanced facilities (58). The heavy reliance on private healthcare is especially problematic in low-resource settings, where associated costs often hit those already struggling the hardest. For instance, a study on older adults in India with disabilities and multiple chronic conditions found that they are particularly dependent on private services, as the public primary care system fails to meet their needs (59).

In 2018, India launched the Ayushman Bharat initiative as a step toward closing persisting healthcare gaps and working toward UHC for its population. This program has two main components: strengthening primary healthcare and providing health insurance coverage. Up to 5 lakh rupees per capita were allocated for secondary and tertiary care for the disadvantaged 40% of the population (49). However, evaluation studies on Ayushman Bharat have shown persistently low health insurance coverage, and even in areas with high enrolment, improvements in service quality and utilization remain limited (60, 61). These implementation challenges considerably limit the program's effectiveness for the disadvantaged populations it aims to support, especially in the low-resource settings of rural areas and urban slums.

### Objectives

Improving healthcare access and service delivery in rural areas and urban slums requires a profound understanding of the health perceptions and healthcare expectations of people living in these areas. To address this need, we conducted a qualitative study in the slums and rural areas surrounding Bengaluru (formerly called Bangalore), South India, exploring the question: How do people in these low-resource settings perceive health and healthcare? This research was carried out in collaboration with Bangalore Baptist Hospital (BBH), a charitable organization dedicated to supporting underserved populations in this region, with the goal of meeting the unique needs of these communities through better healthcare strategies.

## Methods

This study draws upon a descriptive approach to qualitative research, aiming to offer detailed insights into health and healthcare perceptions in a specific setting (62). To this end, we selected open-ended qualitative interviews and focus groups as data collection methods. One-on-one interviews provided detailed insights on individual perspectives, while focus groups facilitated valuable insights into collective attitudes and group dynamics (63). This combination of methods ensured a comprehensive and nuanced understanding of the research topic. It also allowed participants to choose their preferred setting; some were more comfortable in one-on-one interviews, while others favored the group discussions.

### Setting

**Figure 1:**
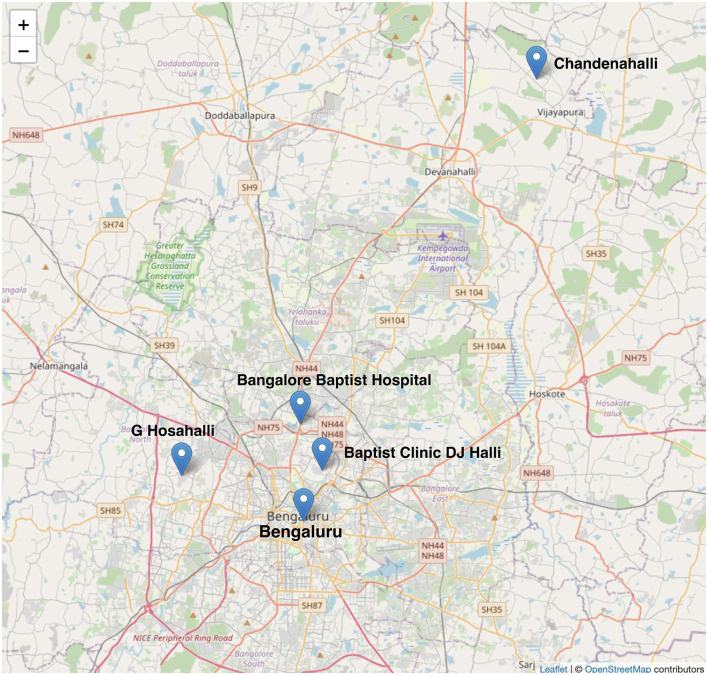
Map of Bengaluru with markers showing the location of Bangalore Baptist Hospital, slum clinic and rural areas where qualitative interviews were conducted. Map data © OpenStreetMap contributors, rendered with Mapnik (64).

The setting was chosen to explore different low-resource settings of urban slums and rural areas in the Indian context. Bengaluru, a city in the southern part of India, is the country's third most populous city, with over eight million residents and a metropolitan population of 15 million. It is one of the fastest-growing cities in India, and ~10% of its population lives in slums. The choice of areas for qualitative research was informed by researcher EG from the Community Health Department of BBH. BBH delivers essential healthcare services to low-income patients across urban and rural areas of Bengaluru. Their outreach efforts include running an outpost clinic in slums and operating mobile clinics in villages that lack hospital facilities. For the research on urban slums, DJ Halli, also known as Devara Jeevanahalli, one of Bengaluru's largest slums, was selected. To capture diverse perspectives based on community and religious differences, one predominantly Muslim area (DJ Halli- 1) and one predominantly Tamil Hindu area (DJ Halli- 2) were included. In rural areas, Chandenalli which is Northeast of Bengaluru and G. Hosahalli which is to the West of Bengaluru city were chosen. All the three areas in relation to the city and BBH can be seen in the map in Figure 1 (64).

### Sampling

To capture a wide range of perspectives on health and healthcare, we employed purposive sampling with a maximum variation approach, selecting participants to ensure diversity in age, gender, and physical location (65). The inclusion criteria required participants to be residents of the selected settings and over 18 years of age. While the study aimed to gather perspectives from the general population, individuals experiencing illnesses at the time were neither specifically sought out nor excluded.

### Recruiting

Recruitment was facilitated by community health workers from BBH. In the villages, additional support was provided by Anganwadi teachers (i.e., village nutrition workers employed by Integrated Child Development Services (ICDS) government program) (66). Twenty-six persons participated in individual interviews this study, including 14 women and 12 men. Nine of them were from predominantly Muslim and four from predominantly Tamil Hindu urban slums and thirteen people living in rural areas were included. Eight focus groups with 5–12 people were conducted. The participants of focus groups were either predominantly men (*n* = 5) or predominantly women (*n* = 3). An overview of participant demographics is presented in Tables 1, 2.

**Table 1:** Participant characteristics—open-ended qualitative interviews.

**Participant no**.	**Age**	**Gender**	**Setting**	**Education**	**Occupation**
1	37	F	DJ Halli- 1	1st grade	Real estate business
2	62	M	DJ Halli- 1		
3	46	M	DJ Halli- 1	Graduate	
4	39	M	DJ Halli- 1	3rd grade	Taxi driver
5	28	F	DJ Halli- 1	8th grade	Housewife
6	42	F	DJ Halli- 1	7th grade	Housewife
7	25	F	DJ Halli- 1	3rd grade	cook
8	33	F	DJ Halli- 1	5th grade	Housewife
9	55	F	DJ Halli- 1	5th grade	Housewife
10	55	F	DJ Halli- 1	6th grade	Housewife
11	35	F	DJ Halli- 1	5th grade	Food business
12	26	M	DJ Halli- 2		Vegetable merchant
13	21	M	DJ Halli- 2	12th grade	finance collection
14	24	M	DJ Halli- 2	Graduate	for US health department
15	65	F	DJ Halli- 2	7th grade	stays at home
16	38	M	Chandenahalli	4th grade	daily labor
17	30	M	Chandenahalli	9th class	daily labor
18	60	M	Chandenahalli	10th class	agriculture
22	36	F	Chandenahalli	7th class	Housewife
23		F	Chandenahalli		Housewife
24		F	Chandenahalli		Housewife
25	18	F	Chandenahalli	12th class	studying
19	40	M	G. Hosahalli	no education	
20		M	G. Hosahalli		Dairy Business
21	62	M	G. Hosahalli	7th class	
26		F	G. Hosahalli		housewife
27	40	F	G. Hosahalli		housewife
28	20	F	G. Hosahalli		housewife

Empty cells indicate missing data where information was not provided by participants.

**Table 2:** Participant characteristics – focus groups.

**Group**	**Setting**	**Age group (years)**	**Gender**
1	DJ Halli- 1	35–60	Men
2	DJ Halli- 1	27–62	Men
3	DJ Halli- 1	21–65	Women
4	DJ Halli- 2	21–55	Men
5	DJ Halli- 2	20–45	Women
6	Chandenalli	32–58	Men
7	G. Hosahalli	36–65	Women
8	Chandenalli	45–67	Men

### Interview and focus group guides

A pilot focus group interview was conducted with individuals visiting Bangalore Baptist Hospital (BBH) as part of a health education program prior to the main data collection. This preliminary study helped refine the discussion guide and interview questions. Additionally, the guide was further adjusted throughout the data collection process based on insights gained from each interview. Each interview began with the question, “How are you feeling today? Are you feeling well?” This simple opening encouraged participants to share their views on what health and being healthy meant to them, setting the stage for further discussions. Discussions on health frequently led to conversations about healthcare. When they did not naturally shift in this direction, participants were encouraged to share personal experiences with HCFs, either for themselves or family members. They were then encouraged to reflect on both the positive and negative aspects of these experiences and to imagine their ideal HCF, describing the features they considered essential for optimal care.

### Data collection and analysis

KD conducted interviews on-site in the slums and villages within the participant's houses and community facilities. A community health worker from BBH, was present at the beginning of most interviews but stepped outside shortly after to attend to her usual duties. Her presence was invaluable in gathering data within this challenging and hard-to-reach research environment. Interviews were conducted in local languages. KD conducted the interviews in Telugu and Hindi independently, while an interpreter assisted with the interviews in Kannada and Tamil.

Focus groups were conducted at Anganwadi schools (i.e., community centers that improve the health and education of young children) (67) and at other community areas such as temples. Open-ended qualitative interviews lasted between 20 and 45 min, while focus groups typically lasted about 1 to 1.5 h. Audio recordings of the interviews and focus groups were collected and analyzed along with the field notes for emerging themes. Data was collected until data saturation was attained and no new themes emerged. Once the data collection was complete, the audio recordings were transcribed and translated to English. The transcripts were analyzed using LibreOffice versions 6.2 and 6.4.2.2 employing a manual thematic analysis approach. Themes were developed by KD through an iterative process of reading, coding, and categorizing the data, in line with established qualitative research practices. In reporting the findings, direct quotes were edited for grammatical accuracy while preserving their original meaning. All transcripts are publicly accessible through the research data repository Mendeley Data (68).

### Ethical consideration

Ethical approval was granted by the Institutional Review Board of BBH, which is recognized by the University of Sheffield. The research was approved by the University of Sheffield as part of the KD's master's thesis. In accordance with the Data Protection Act 2018, which incorporates the General Data Protection Regulation into UK law, as well as its Indian counterpart, the Human Resource Accounting Policy Framework 2018, all collected data was stored securely. A participant information sheet detailing the research objectives, privacy considerations, confidentiality measures, and potential risks and benefits was prepared. It was read out and thoroughly explained to participants. Informed written or verbal consent was taken from all the participants before the interviews and focus groups.

### Researcher positionality

KD's background in clinical medicine, and prior experience working with underserved populations in India provided her with a strong understanding of healthcare in the low-resource settings under study. This background also helped them manage their emotions while conducting research on a sensitive topic with a marginalized group (69). Having grown up in India, KD shared a cultural background with some participants, which facilitated trust and helped build rapport. However, this also posed a risk of making incorrect assumptions about mutual understanding.

To address this, several measures were implemented. During interviews and focus groups, KD engaged in careful probing and sought clarification to avoid misinterpretations. She also maintained a reflexive journal to document assumptions, biases, and decisions and regularly engaged in discussions with colleagues to critically evaluate alternative interpretations of the data. These steps ensured that the findings accurately reflected the participants' voices while minimizing the influence of the researcher's own perspective.

To enhance transparency and rigor in reporting, a completed COREQ checklist (70), along with the interview guide is provided in Supplementary material.

## Results

### Perception of health

Participants were asked if they thought they were healthy, and what being healthy meant for them. Some participants perceived the latter question rather as being asked about how to be healthy, generating rich insights into their perception of health. Data analysis resulted in three main themes: (1) Absence of illness, (2) Ability to work (3) Result of a good lifestyle.

1. Absence of illness

While some participants viewed health as the absence of illness (Q1), others made a distinction between temporary and chronic health problems. For some, temporary and acute issues like a cold or fever were not considered health problems (Q2). Meanwhile, others viewed themselves as healthy despite having a chronic condition like diabetes but regarded temporary issues as health problems (Q3).

“To not have any problems, to not have things like leg pains and eye pains, I don't have anything right, that is healthy.” Participant 20- Q1

“to be healthy means - fever and all is fine, to not have any other problems is healthy.” Participant 1- Q2

“Interviewer: What is health according to you?

Interviewee: Health is to not have fever, headache etc, I have diabetes,

Interviewer: So, you have diabetes, would you say you are healthy?

Interviewee: Yes, I am healthy” Participant 18- Q3

Participants described health as having peace of mind and acknowledged that stress can lead to health problems (Q4). However, when asked about mental health issues, they were often unaware of the concept or uncomfortable discussing it, stating they did not know anyone with such issues in their communities.

“important thing is that the mind is free, there should be no tension and only then we will be healthy. If there are tensions, nothing is good, and you will get sick. Mainly people will get sick from all the tensions.” – Focus group 3 - Q4

2. Ability to work

Health was often defined in the context of work. Most participants considered health as the ability to work (Q5, 6). Work-related health was both related to paid labor and to unpaid household work, including the ability to take care of family, friends, and others (Q7).

“Being healthy means, we are fine, we are fresh, there is no pain, we are able to do our work, that is being healthy.” Focus group 3 – Q5

“Healthy is when our body is free and we are able to work, that is being healthy. If we cannot work properly, then there is something wrong with our health.”-Focus group 8- Q6

“Being healthy is the best thing for everything, to look after your family, friends or around you. In this world, if you are healthy, only then you are useful. If you are not healthy, then you are useless,” Participant 3 - Q7

Moreover, residents of the villages stated that they consider themselves healthier than those in the cities due to the physical activity involved in their daily work (Q8).

“In cities, they are always sitting right? They get a lot of problems. We are doing a lot of work, and we are always moving. We only get things like fever occasionally. We are mostly healthy” Focus group 7 -Q8

3. Result of a good lifestyle

Participants viewed health as something resulted from a good food-, work- and sleep-related lifestyle (Q9). Eating well (Q10) and digesting well (Q11) was considered as a particularly important component for good health, especially by older people. As in the context of work, residents in the villages considered themselves healthier than those in the cities due to, what they considered, better eating habits (Q12).

“What is health… eating well, working well, so, we can sleep well. In that sense we are healthy.” Focus group 5– Q9

“Health is, it's in the food we eat. If people don't work and eat sweets and rice, you will get diabetes and all. If you eat ragi mudde (a nutritious finger millet dumpling dish which is a staple food in these regions), and work well, you won't get any problems.” Focus group 6 – Q10

“If you don't have body pains or any other pains, and if you eat well, on time, and digest it well, that is health.” Participant 15- Q11

“We eat ragi mudde in villages, we don't get problems as much as city people. We have problems, but not much.” Focus group 6- Q12

Along with eating well, young participants acknowledged the importance of physical exercise beyond their daily work (Q13).

“Health is having three meals a day, doing some exercise and keeping muscular. If someone is unhealthy, the food they eat matters and maybe they are not getting good proteins or nutrition.” Participant 12- Q13

### Healthcare expectations

Participants were aware of the various public and private HCFs available to them. Some also mentioned visiting traditional medicine practitioners for certain ailments, though this was not a common practice among the participants in this study. However, when asked about their views on existing healthcare services and the changes needed to better meet their needs, participants struggled to answer. Some said they cannot change anything, noting things were the way they were and that what they wanted did not matter. Participants were then asked to describe their previous experiences with HCFs—what they liked and disliked, and why they go to the HCFs that they go to. This initiated longer replies and generated valuable insights into their healthcare expectations. Data analysis resulted in six main themes: (1) Good Doctor, (2) Getting well quickly, (3) Affordable costs, (4) Cleanliness, (5) Emergency services and diagnostic facilities, and a theme highlighting the stigma against seeking medical help, namely (6) “I do not go to a hospital”.

1. The “good doctor”

Participants stated that having a “good doctor” was the most important aspect of a HCF for them (Q14).

“In this area, he is the only good doctor, gives good treatment, the medicines suit us, we get cured in 2 days.” Participant 1- Q14

In Telugu, some participants expressed their overall satisfaction with their doctor as “baga chustharu”- which can be translated as “they treat us well”. Participants interpreted what it means to be a good doctor in different ways. For some participants, it merely meant that the doctor restored their health through providing treatment and medicine (Q14). For most, this meant being treated kindly and respectfully, extending beyond just receiving medical care in terms of prescriptions. Participants valued good communication with their doctors, wishing for them to speak softly, understand their concerns, answer their questions, and treat them like friends (Q15). The way doctors attended to them, combined with their ability to restore health, helped build participants' trust in them (Q16). Some participants even believed that calming conversations between doctors and patients had a positive impact on their health issues on their own (Q17).

“First, as the patient goes to the doctor, they should speak softly. The doctor should ask them what their problem is. If the doctor speaks a little loudly, the patients won't go there. They will say ‘no no, I don't want to go to that hospital'. If the doctor asks what their problem is, like friends, we would be open to share our problems. As soon as patients enter, they should be offered to sit down and treated with respect. If they do that, the patients will automatically go there. Facilities are important, but the doctor should ask ‘what's your problem?' etc., like friends, then patients will go to them.” Participant 23– Q15

“Doctor should talk well, and give good medicines, that's why we go there. Some doctors – they just give an injection and send you away, and some doctors, they ask everything, how did it start, when did It start and ask all these things, that's a good doctor. Curing us and talking well, both are important, we get the trust that the doctor gives good treatment”- F-VM-1 – Q16

“First thing is they should talk straight to the patients. Straight means in a nice way, with respect. When someone comes with a pain, you should not be irritated by them, you should talk to them in a relaxed way so that patient can share their problem. A good conversation like this can take away half of our problems”- SMW-1 – Q17

Participants placed little emphasis on doctors' formal qualifications. Instead, they assessed a doctor's competence based on factors such as the speed of their recovery, the quality of care received, and recommendations from acquaintances (Q18, Q19).

“(When going to a new hospital), we do not know if the doctor is qualified. We go and ask in a big hospital that we know – about this new hospital. And we ask our friends and neighbours who have been to this new hospital for their opinion.” Participant 20 – Q18

“People who are educated check the certificate (in the doctor's room). People who are not, only care about getting cured quickly, for them, the important thing is for the doctor to see them well” Focus group 7- Q19

Moreover, the persons interviewed in this study preferred “good doctors” over advanced technical equipment. They valued attentive interactions with doctors who understood their health concerns and effectively treated their illnesses more than diagnostic tests or well-equipped HCFs (Q21, Q22).

“If the doctor is good, I can get the tests (i.e., diagnostic tests) done somewhere else. If the doctor is not good, what do I do with the tests? What is the value?” Focus group 6- Q21

“Interviewee: that clinic is right here. It's been 20 years since we came to this area. from the past 20 years has been going to the same doctor same place, the doctor is very good, and he gives good medicine for everyone. the treatment is very good. people come from a lot of places because he's very good. With other hospitals, you need two or three appointments to recover, here you go once, and you will be cured.

Interviewer: What do you like about it? Can they admit patients? Do they have beds?

Interviewee: No, it is a small clinic there is not much space.

Interviewer: Other facilities for investigations (i.e., diagnostic tests)?

Interviewee: No there is nothing, we must go to some other private places for investigations maybe a close by clinic or some other Private Investigations, which are far away.

Interviewer: If I ask you about things lacking in this clinic, what would you say? How can it be improved?

Interviewee: Nobody will say anything about it. Everyone likes it.” Focus group 3- Q22

2. Recovering quickly

While achieving recovery was a key expectation from healthcare among participants (Q23), the time and efficiency of the receiving treatment were equally important (Q24). Participants valued prompt improvement and placed importance on various time-related factors, both within and outside HCFs, such as the number of required visits (Q25), waiting times, duration of tests, commuting time, and all aspects involved in the journey from illness to recovery (Q26). Additionally, having continuity of care, rather than changing doctors on different HCF visits, was important to them for a quicker recovery, as it prevented the need to repeatedly explain their condition and redo tests (Q26).

“According to me, good hospital means, a person should get cured, So, that will be a good hospital. If he is not cured, then the hospital is not good” Participant 3-Q23

“They take less money in other places, but the treatment doesn't suit us, the medicine doesn't affect us immediately and we are not cured immediately. Here, even if they take a bit more money, you get cured immediately. That's why we go.” Participant 8- Q24

“With other hospitals, you need two or three appointments to recover, here you go once, and you will be cured.” Focus group 3- Q25

“But they (i.e. the staff at one government hospital) just make us go around. They tell us to bring this report and that report. In an entire week, your doctor is there only for one day. If your initial consultation is on Wednesday, you can revisit only on Wednesdays. If you have a problem during the week, what do you do? If you go to the hospital, you will not find the doctor, your doctor. In that case we must consult another doctor and start from the beginning again. We must show the form again, and we must do the test again, so we got tired and gave up.” Focus group 3 -Q26

Furthermore, recovering quickly was closely tied to monetary factors (Q27). Participants often compared slower public hospitals with faster and expensive private ones (Q27, Q28). Participants also perceived some diagnostics and other procedures at government hospitals to be “in-the-way” of their treatment, and preferred private facilities that came straight to prescribing medicines (Q28). They also noted that out-of-pocket spending could accelerate treatment for those who could afford it. For instance, one slum resident mentioned that services in a government hospital are quicker only if money is offered (Q29).

“We got so tired, and my kid was not getting better. We had to roam around a lot for tests and everything. We had to wait for everything, and they were just telling us to wait. Because we did not have money we had to go to government Hospital, and, we have a card (subsidy) which is useful in government Hospital.” Focus group 3 –Q27

“When one goes to the government hospital, doctors don't see you immediately. Staff will say that the doctor isn't in yet and you must wait forever. If you go to a private hospital, even if they charge you more, the relevant doctor will see you immediately and the treatment will happen immediately. If you go to a government hospital, they will say things like ‘go and get this test done' and ‘your sugar should be normal your blood pressure should be normal to continue with the next steps', there are all kinds of things delaying treatment. In the private hospital everything happens so quickly” Focus group 3 – Q28

“Only if we give money to some people (i.e., some staff members at the government hospital), they will see us quickly. If a poor person goes there, he must roam around a lot, they tell them to go here, and then there.” SMW – 3 – Q29

3. Cost affordability

While some participants indicated that they only went to government hospitals out of financial necessity (Q30), others focused on the value of receiving free treatment in government hospitals (Q31).

“In the government hospital, they give free medicine, that is convenient for us. In private hospitals, we must pay” Participant 17-Q30

“They don't take money; they are best” Focus group 2 – Q31

Several participants factored in expenses beyond treatment and medication when explaining their preference for certain HCFs. Despite the lower costs at public government facilities, they expressed a negative attitude toward them. This was due to additional travel costs that could be avoided by visiting a nearby private hospital, as well as the belief that they needed more frequent visits to government facilities to achieve the same level of care as at a private hospital (Q32, Q33).

“About the cost (at a private clinic), for one injection, the doctor takes 800 rupees. They should not take so much; they should take a little less. With our incomes, we cannot afford this. You need to spend at least 500 rupees per visit. Even for cold and fever, we must pay 500 rupees. For poor people like us, they should charge less. We run to them for better cure, we don't want to worry about money, we just want the kids to recover. If we go to government hospital, they make us run around for days and they don't give any treatment, and the auto charges are already 20 rupees per trip one-way” Focus group 3- Q32

“They prescribe medicines that we have to buy (instead of the medicines one can get for free at government hospitals) from elsewhere and altogether it costs us a lot we might as well go to a private hospital which is next to us, instead of going to the government hospital and waiting and taking all the trouble,” Focus group 7-Q33

4. Cleanliness

Cleanliness was another key factor that mattered to participants. For some, cleanliness in HCFs was important for maintaining hygiene and health (Q33, Q34), while for others, it contributed to their comfort and overall positive experience at the hospital (Q35).

“In some (govt) hospitals, toilets are not clean, they are disgusting. It should not be like that. They should be clean.” Participant 22-Q33

“In a good hospital, neatness should be good. See, a lot of people come to the hospital with a lot of diseases. There are all kinds of diseases. When one patient gets discharged, they just change the sheet, but the pillow cover stays the same for the next person. That's not good for the new patient, right?” Participant 4-Q34

“They should reduce the number of patients (in govt hospitals) and keep it clean, like move some patients to other wards. Otherwise, there are a lot of patients in the same ward, there is no comfort for the patient.” Participant 5-Q35

5. Emergency services and diagnostics facilities

Participants expressed the need for emergency services (Q36, Q37), which are accessible and affordable to them in terms of distance and time (Q38). Despite their closer proximity to HCFs, residents of slums emphasized more on shortcomings with regards to emergency services than those living in villages. Notably, some participants from villages expressed satisfaction with their available resources. They mentioned having access to the 108-ambulance service (71). private transportation options, and a supportive community that helps during times of need (Q39).

“There is a need for a good hospital where the treatment is good. If there is an emergency, and the patient could die in 10 or 15 min without treatment, there are no facilities for patients like that. For accidents and such, there are no good facilities.” Participant 4-Q36

“For emergencies, and the person needs to be saved in 30 minutes, there is no emergency facilities. To go anywhere, it takes one, one and a half hours.” Participant 17 – Q37

“For the sake of emergency there should be something. And the one that is close by, does not fit with the budget. We must consider whether we have enough money before going there.” Participant 5-Q38

“(When someone is not well), they can't travel by bus right? […] We ask our neighbors, tell them it is an emergency and take their help if they have a vehicle. […] We ask someone and manage somehow. There is a unity in villages, people help each other. People are willing to help each other. Also, we don't have many emergencies, it is usually manageable. When there is an emergency, then we call 108. 108 has been very useful and convenient. For deliveries (childbirth), if there is no one around, 108 is the only option” Focus group 7- Q39

Persons interviewed for this study expressed the need for comprehensive diagnostics facilities within their HCF, so that they do not need to visit other facilities only for taking the medical tests (Q40, 41, 42). Some participants with knowledge of medical procedures expressed a desire for comprehensive diagnostic facilities equipped with modern technology (Q43).

“See, why have we been sent to some other hospital for a head X-ray and everything? Because they are not available here. People get their hands and legs broken, that is more (common) here.” Focus group 2-Q40

“There should be machines to check BP (i.e. blood pressure), sugar, and scanning should be there, ECG (i.e. an electrocardiogram) should be there and all these main things should be there.” Participant 16-Q41

“It's better in big hospitals, all the equipment is at one place. We can go from one department to another and just get the tests done there. Everything is at one place. It's not the same with the clinic (i.e. private clinic that he goes to and likes otherwise). Here they only give the requisition, you must go somewhere some other place and get the tests done” Participant 4-Q42

“The hospital should have the facilities, what you call, hi-tech facilities, like operation theatres, and tests (i.e. diagnostics), ultrasound, and MRI scanning, those facilities should be there.” Participant 3-Q43

6. “I don't go to a hospital”

Some participants from villages also expressed a negative attitude toward seeking medical care in hospitals. Some interviewees cited feeling healthy as the reason for avoiding medical tests, believing that abnormal results would cause unnecessary worry. Moreover, participants avoided hospital visits due to concerns that their community might view them negatively, reflecting the stigma associated with illness (Q44, Q45).

“See, I know that if we take tablets, it will be okay, some people don't know. Now BP, blood sugar and all, 80 percent people have it, only 20 percent don't, even small kids have these things these days. Some people know about this stuff, others don't, they will fear what people say. We feel delicate about such things. We think ‘if I go to a medical camp or the mobile clinic and say I have a problem, other people in my village will get to know about my problems as well'.” Focus group 6 -Q44

participant “a” - “I can't see small letters, and when I wear specs, I can. You know what people say if I wear specs, that I am blind (laughing). Or they say that I am showing off my style (previously wearing glasses was seen as a fashion statement, and in rural areas, it was often associated with movie stars or wealthy city people. Among older people, this association still seems to hold),

participant “b” – Yeah me too, I can't see small letters, I also need specs, I have been ignoring this for 2 years.

participant “c” - “In villages, people comment, they say ‘see that man, he is showing off with his glasses at this old age'. It is hard to explain things to you. This is a village, this culture is different from cities, other people don't know our culture. People here tend to pass comments at each other” Focus group 6- Q45

## Discussion

This study aimed to understand perceptions health and healthcare among people living in rural areas and slums around Bengaluru. Participants indicated several perceptions of health, including, the absence of illness. Some participants mentioned that experiences of stress can affect health, but mental health was not talked about much. In addition to absence of illness, health was often related to the ability to work and having a good lifestyle and a good diet. These views are similar to previous research on lay perceptions of health (3, 5, 8).

With respect to their perceptions on healthcare, consistent with findings from similar studies, this study shows that people perceive medical care majorly as their interaction with the doctor (6, 28–30). Participants expressed a strong preference for good communication and respect from their doctor over other factors. Studies have shown that a positive relationship with healthcare providers is a key factor in people-centered care (29, 72, 73). As seen in previous research, participants in this study gave more weight to testimonials from their friends, family, and other acquaintances rather than the qualifications of the doctor (29). This could help explain the popularity of clinics run by unqualified medical practitioners (44, 74). While policymakers focus on providing qualified doctors and facilities, people seem to care more about soft skills (29). People's expectations should be met in these areas to provide a healthcare that satisfies the definition of “quality” for both healthcare providers and people.

Participants emphasized that affordability is another important factor they consider when seeking healthcare. However, despite the lower costs associated with public healthcare, they largely preferred private healthcare. The inadequacy and underutilization of the public healthcare in India in terms of capacity and quality and associated out of pocket expenditures are well-documented (44, 53, 56, 75). As found in previous studies (24, 53, 56), participants did not only associate affordability with the direct costs of healthcare services but also with additional expenses, including travel costs, loss of daily wages, and the time required for the entire process. They also expressed concerns about the quality of treatment in public HCFs.

Participants said that a quick recovery is important for them. As seen in previous studies, travel, waiting times, and anything that increases the time span of “illness” is undesirable (76). This is not only because they are inconvenient, but also because they result in lost work hours and, consequently, lost wages (77–79). This is related to health perceptions. Many participants viewed health as the ability to work, and judged HCF efficiency based on how quickly they could return to a healthy state. In this regard, participants perceived diagnostics and other procedures at government hospitals as obstacles to treatment, preferring private facilities that directly prescribed medications. On the other hand, previous research highlights the use of unnecessary and inappropriate medications by private practitioners in India, to satisfy patient's expectations (76). The study found that patients preferred these medications over going through diagnostics and making multiple trips to the (usually government) hospital. This preference may reflect their generally unsatisfactory experiences at government hospitals. These findings suggest a need to improve health literacy regarding the importance of appropriate medical procedures, potentially strengthen regulation of private HCFs, and ensure that healthcare services account for the practical time constraints patients face.

Similar to research on other low-resource settings (21, 80, 81), this study found cleanliness of HCFs to play a key role in healthcare seeking behavior, with sanitation at HCFs influencing patient satisfaction.

While residents in urban slums and rural areas provided similar perspectives on health and healthcare, this study also found differences between these populations. Both groups expressed the need for accessible emergency services. However, the reasons for that wish differed due to different healthcare infrastructures available to them as well as financial constraints, as was also found in previous studies on India (43, 44). For those in villages, distance was the primary barrier, while for residents of slums, affordability was the main constraint to emergency services, despite the availability.

Furthermore, this study identified fear of being stigmatized when seeking healthcare among people in villages, as well as a lack of awareness about health issues, both of which was not indicated by participants from urban slums in this study, but found for this population in earlier research on slums in Bengaluru (82). Despite these potentially detrimental factors to health outcomes, participants from villages described themselves as being healthier than urban populations, emphasizing their healthy food habits and physical daily work. Similarly, discrepancies between self-perceived health status and medical advice—evident in associations between higher self-perceived health and behaviors like drinking and higher BMI—highlight the need for health education targeting these risk factors (17). While positive health perceptions can be associated with improved health outcomes (6, 9, 14, 15), the existing disadvantages in healthcare access and outcomes for people in villages need to be addressed.

### Limitations

Like other qualitative studies, this research is limited in its ability to represent the broader population. Further quantitative research is necessary to enable the generalization of these findings and their application in diverse contexts. Data collection for this study took place under challenging conditions due to the low-resource research setting in urban slums and rural areas in India. While collaboration with local community health workers facilitated data gathering, the research design had to adapt to these circumstances. For example, it was not possible to conduct entirely confidential focus group discussions, as other people such as family and neighbors were often present during individual interviews and focus groups. Although the data collected from the focus groups was generally of good quality, the presence of bystanders may have influenced some of the conversations. Additionally, interviews were only conducted during the day (working hours of accompanying community health workers) for practical reasons. This limited men's availability, as they were often engaged in paid labor during daytime hours. This issue was more pronounced in slums than in villages, where men's workplaces were typically closer to their homes, making them more accessible for interviews.

Moreover, the analysis showed neither significant differences between men's and women's perceptions of health and healthcare nor between those of Muslim and Tamil Hindu participants. In low-resource settings in India, women's agency in healthcare decisions is often limited by the need for approval from a key male household member (39). We expect that research focusing on gender dimensions can help in understanding this situation's implications for healthcare services and its broader impact. This study selected one area with predominantly Muslim and one area with predominantly Tamil Hindu populations, yet found no differences, despite the socio-economic disadvantages faced by Muslims in Bengaluru's slums (83). This may be due to recruitment through BBH community health workers, who could only introduce individuals already connected to this informal healthcare structure.

### Policy and practical implications

This study highlighted key areas where healthcare access and trust could be improved for residents of slums and rural areas in and around Bengaluru. Policymakers could adopt a more comprehensive understanding of healthcare costs, recognizing that financial burdens extend beyond direct expenses to include travel costs and lost wages. Expanding mobile healthcare units in rural areas, improving scheduling systems in government hospitals to reduce wait times, and exploring compensation models—such as wage reimbursement for daily laborers accessing care—could help mitigate these hidden costs. Improving Public healthcare infrastructure in specific areas, particularly cleanliness, service efficiency, and waiting times, could be of great value, as these factors seem to influence healthcare-seeking behavior. Community-based health literacy interventions could further support access to care by increasing awareness of appropriate medical procedures such as diagnostics and address the limited discussion of mental health observed in this study. Additionally, efforts to improve access to emergency care may also benefit from approaches tailored to different challenges. In rural areas, where distance is a key barrier, expanding ambulance services could enhance emergency response. In urban slums, where financial constraints are a greater concern, making emergency care more affordable through subsidies or public-private partnerships may improve access. Given participants' reliance on private healthcare providers, strengthening regulations on private facilities, particularly in ensuring ethical prescribing practices and monitoring unqualified practitioners, could contribute to more consistent and safe patient care.

Healthcare providers and institutions also have an important role in addressing concerns around trust and treatment experiences. Since participants in this study emphasized the importance of good communication and respectful interactions with doctors, professional training in empathetic communication may help improve provider-patient relationships. Implementing patient feedback mechanisms in clinics and hospitals may help address concerns and improve the quality of care. In public healthcare facilities, clearer explanations of medical procedures, particularly diagnostics, could help address the perception that such steps are unnecessary obstacles rather than essential components of care. Attention to hospital cleanliness, efficiency, and patient dignity may also contribute to improving trust in public healthcare services.

Community organizations and NGOs could support healthcare access and literacy through locally driven initiatives. Peer-led health education programs, where trained community members raise awareness about preventive care, mental health, and the importance of proper diagnostics, might help bridge gaps in health knowledge.

Although these recommendations are based on the specific context of this study, further research could explore ways to adapt and implement similar strategies in other low-resource settings.

## Conclusion

This study explored the health and healthcare perceptions of people living in slums and rural areas in and around Bengaluru. The results complement previous research on lay perspectives in low-resource settings in India and elsewhere. They identify areas of improvement for designing better healthcare systems for people living in these areas.

Participants emphasized the importance of good relationships with their healthcare providers and geographically accessible HCFs. To improve the uptake of healthcare services among residents of slums and rural areas in and around Bengaluru and attain UHC, healthcare facilities in these areas need to focus on improving professional interactions, reducing waiting times and improving health literacy. Another key aspect was affordability. While the newly introduced Ayushman Bharat public insurance program covers direct healthcare costs, additional out-of-pocket expenses such as travel costs and loss of wages when accessing healthcare persist. This highlights the need to develop an understanding of healthcare costs as a comprehensive expense, rather than focusing only on the direct medical expenses.

By recognizing both structural barriers and the nuanced realities shaping healthcare-seeking behaviors, these strategies could contribute to more equitable and effective healthcare delivery. Future research could further explore how such approaches might be adapted to different contexts, ensuring that healthcare improvements are responsive to local needs.

## Data Availability

The datasets presented in this study can be found in online repositories. The names of the repository/repositories and accession number(s) can be found in the article/Supplementary material.
