# Supplementary material for: Perceptions of health and healthcare needs in low-resource settings: qualitative insights from Bengaluru's urban slum and rural areas
Source: Front Public Health. 2025 Apr 1;13:1530256. doi: 10.3389/fpubh.2025.1530256 (PMC11996843; doi:10.3389/fpubh.2025.1530256)
Supplement: Supplementary file 1 [file Data_Sheet_1.docx]

**Guide for Individual interviews and Focus groups**

1. As of today, are you and everyone in your family healthy? (if not, what is wrong?) – opening question that everyone can answer – a factual question
2. How do you define ‘good health’ or ‘being healthy’?
3. How many times did you go to a healthcare facility or health advice in the past 3 months? If they haven’t been to a healthcare facility in the past 3. Months,
4. How many times did you go to a healthcare facility in the past 6 months?

(introductory questions – which helps people reflect on the past experiences and connect with the topic)

1. For what issues did you go to the hospital in the past 3/6 months?
2. And where (which facility) did you go to in these instances?

Key questions – these questions require the greatest attention in analysis

1. What exactly happened when you went the last time?
2. Why did you prefer these facilities over others? Details about each facility individually
3. Are you satisfied with the treatment /care you received in these facilities? Details about each facility individually
4. Let’s make a list of things you like and don’t like about each of these facilities
5. What do you think is an ideal healthcare?

More transition questions

1. What kind of illnesses do people around your neighborhood have? What do you usually see?
2. How do you feel about the amount or number of facilities available around here? Are there enough? Too little? (transition question – serves as a link between introductory and key questions)
3. To fit the needs of the people living here, what modifications should be done to these health care facilities?
