# Supplementary material for: Perceptions of health and healthcare needs in low-resource settings: qualitative insights from Bengaluru's urban slum and rural areas
Source: Front Public Health. 2025 Apr 1;13:1530256. doi: 10.3389/fpubh.2025.1530256 (PMC11996843; doi:10.3389/fpubh.2025.1530256)
Supplement: Supplementary file 2 [file Data_Sheet_2.docx]

**COREQ (Consolidated Criteria for Reporting Qualitative Research) Checklist**

Developed from:

Tong A, Sainsbury P, Craig J. Consolidated criteria for reporting qualitative research (COREQ): a 32-item checklist for interviews and focus groups. *International Journal for Quality in Health Care*. 2007. Volume 19, Number 6: pp. 349 – 357

**Title of Study:** “Perceptions of Health and Healthcare Needs in Low-Resource Settings: Qualitative Insights from Bengaluru’s Urban Slum and Rural Areas”
**Authors:** Keerthi Dubbala, Wanda Spahl, Carolin Elizabeth George and Luc de Witte

| **No. Item** | **Guide questions/description** | **Details (as needed)** |
| --- | --- | --- |
| **Domain 1: Research team and reﬂexivity** |  |  |
| *Personal Characteristics* |  |  |
| 1. Inter viewer/facilitator | Which author/s conducted the interview or focus group? | Author KD |
| 2. Credentials | What were the researcher’s credentials? E.g. PhD, MD | MBBS, MPH |
| 3. Occupation | What was their occupation at the time of the study? | Master’s student |
| 4. Gender | Was the researcher male or female? | Female |
| 5. Experience and training | What experience or training did the researcher have? | The researcher received formal training in qualitative research as part of their master's coursework. Additionally, they gained practical experience under the guidance of the author EG, from the Community Health Department at Bangalore Baptist Hospital, who had conducted qualitative studies with this population previously. |
| *Relationship with participants* |  |  |
| 6. Relationship established | Was a relationship established prior to study commencement? | A brief introduction of the researcher and the research were given before the interviews and Focus groups. Trust was also created by the presence of a local community health worker who had previously established relationships with participants. Discussed under ‘Researcher Positionality’ and ‘Recruiting’ in ‘Methods’ section. |
| 7. Participant knowledge of the interviewer | What did the participants know about the researcher? e.g. personal goals, reasons for doing the research | Before the research began, the researcher explained their goals to the participants in simple terms, emphasizing that the research aimed to improve healthcare services in the area. Additionally, the researcher read aloud the participant information sheet and addressed any questions. Discussed under ‘Researcher Positionality’ in ‘Methods’ section. |
| 8. Interviewer characteristics | What characteristics were reported about the inter viewer/facilitator? e.g. Bias, assumptions, reasons and interests in the research topic | Discussed under ‘Researcher Positionality’ in ‘Methods’ section. |

| **Domain 2: study design** |  |  |
| --- | --- | --- |
| *Theoretical framework* |  |  |
| 9. Methodological orientation and Theory | What methodological orientation was stated to underpin the study? e.g. grounded theory, discourse analysis, ethnography, phenomenology, content analysis | Theoretical approach is discussed under ‘Introduction’ and methodological orientation is discussed under ‘Methods’ section. |
| *Participant selection* |  |  |
| 10. Sampling | How were participants selected? e.g. purposive, convenience, consecutive, snowball | Discussed under ‘Sampling’ section |
| 11. Method of approach | How were participants approached? e.g. face-to-face, telephone, mail, email | Discussed under ‘Recruiting’ and ‘Data collection and Analysis’ sections. |
| 12. Sample size | How many participants were in the study? | Discussed under ‘Recruiting’ section |
| 13. Non-participation | How many people refused to participate or dropped out? Reasons? | When approached, only two people declined to participate due to work or time constraints. Overall, recruitment was straightforward, and participants were willing and eager to engage in the conversation. |
| *Setting* |  |  |
| 14. Setting of data collection | Where was the data collected? e.g. home, clinic, workplace | Discussed under ‘Data collection and Analysis’ section |
| 15. Presence of non-participants | Was anyone else present besides the participants and researchers? | During the research, community health workers from Bangalore Baptist Hospital, interpreters, and participants' family members or neighbors were sometimes present. Discussed under ‘Data collection and Analysis’ and ‘Limitations’ sections. |
| 16. Description of sample | What are the important characteristics of the sample? e.g. demographic data, date | Discussed under ‘Recruiting’ section. Tables 1 and 2 provide an overview of participants' demographics. |
| *Data collection* |  |  |
| 17. Interview guide | Were questions, prompts, guides provided by the authors? Was it pilot tested? | A pilot focus group was conducted to test the guides. Interview guides are attached as additional files and briefly discussed under ‘Interview and focus group guides’ section. |
| 18. Repeat interviews | Were repeat inter views carried out? If yes, how many? | Repeat interviews were not conducted, in line with the one-time study design. However, the open interview format allowed the researcher to pose follow-up questions during and after the interview. |
| 19. Audio/visual recording | Did the research use audio or visual recording to collect the data? | Audio recordings of the interviews and focus groups were collected after taking consent from the participants. Discussed under ‘Data collection and Analysis’ section. |
| 20. Field notes | Were ﬁeld notes made during and/or after the interview or focus group? | Field notes were made during and immediately after the interviews and focus groups. Discussed under ‘Data collection and Analysis’ section. |
| 21. Duration | What was the duration of the inter views or focus group? | Discussed under ‘Data collection and Analysis’ section |
| 22. Data saturation | Was data saturation discussed? | Data was collected until data saturation was attained and no new themes emerged. Discussed under ‘Data collection and Analysis’ section. |
| 23. Transcripts returned | Were transcripts returned to participants for comment and/or correction? | Transcripts were not returned to the participants; the documented points and field notes were discussed and clarified during and at the end of the interviews and focus groups to ensure accurate representation of the participants’ views. |
| **Domain 3: analysis and ﬁndings** |  |  |
| *Data analysis* |  |  |
| 24. Number of data coders | How many data coders coded the data? | Coding was conducted by author KD, with ongoing discussions and refinements in collaboration with authors EG and LW to ensure rigor and consistency in coding. |
| 25. Description of the coding tree | Did authors provide a description of the coding tree? | No, the manuscript does not include a description of the coding tree. |
| 26. Derivation of themes | Were themes identiﬁed in advance or derived from the data? | The two major themes—'Participants’ views on health' and 'Participants’ views on healthcare'—were identified in advance based on the study’s objectives. However, sub-themes were derived inductively from the data collected during participant interviews. |
| 27. Software | What software, if applicable, was used to manage the data? | No qualitative data analysis software was used to manage the data. LibreOffice was used to organize digital transcripts. |
| 28. Participant checking | Did participants provide feedback on the ﬁndings? | In later interviews and focus groups, after completing the discussion, participants were presented with preliminary findings from earlier analyses and invited to provide their feedback. |
| *Reporting* |  |  |
| 29. Quotations presented | Were participant quotations presented to illustrate the themes/ﬁndings? Was each quotation identiﬁed? e.g. participant number | Key findings of this study were supported with selected quotes in text, along with participant numbers (Results section). |
| 30. Data and ﬁndings consistent | Was there consistency between the data presented and the ﬁndings? | All findings were derived from the data and all themes are supported by illustrative quotes (Results section). |
| 31. Clarity of major themes | Were major themes clearly presented in the ﬁndings? | Major themes from the study design and respective minor themes derived from the data and are clearly defined by paragraph titles (Results section). |
| 32. Clarity of minor themes | Is there a description of diverse cases or discussion of minor themes? | Less commonly discussed perspectives on healthcare, such as the subtheme "I do not go to the hospital," were also identified and explored in the analysis (Results and Discussion sections). |
